# Role of Advanced Glycation End-Products and Oxidative Stress in Type-2-Diabetes-Induced Bone Fragility and Implications on Fracture Risk Stratification

**DOI:** 10.3390/antiox12040928

**Published:** 2023-04-14

**Authors:** Guido Cavati, Filippo Pirrotta, Daniela Merlotti, Elena Ceccarelli, Marco Calabrese, Luigi Gennari, Christian Mingiano

**Affiliations:** Department of Medicine, Surgery and Neurosciences, University of Siena, 53100 Siena, Italy

**Keywords:** type 2 diabetes, fractures, osteoporosis, bone, AGE, RAGE, oxidative stress, reactive oxygen species, antioxidant

## Abstract

Type 2 diabetes (T2D) and osteoporosis (OP) are major causes of morbidity and mortality that have arelevant health and economic burden. Recent epidemiological evidence suggests that both of these disorders are often associated with each other and that T2D patients have an increased risk of fracture, making bone an additional target of diabetes. As occurs for other diabetic complications, the increased accumulation of advanced glycation end-products (AGEs) and oxidative stress represent the major mechanisms explaining bone fragility in T2D. Both of these conditions directly and indirectly (through the promotion of microvascular complications) impair the structural ductility of bone and negatively affect bone turnover, leading to impaired bone quality, rather than decreased bone density. This makes diabetes-induced bone fragility remarkably different from other forms of OP and represents a major challenge for fracture risk stratification, since either the measurement of BMD or the use of common diagnostic algorithms for OP have a poor predictive value. We review and discuss the role of AGEs and oxidative stress on the pathophysiology of bone fragility in T2D, providing some indications on how to improve fracture risk prediction in T2D patients.

## 1. Introduction

Type 2 diabetes (T2D) and osteoporosis (OP) are chronic disorders of glucose and bone metabolism, respectively, with an increasing impact in terms of morbidity, mortality, and healthcare costs, particularly in the elderly population. The increasing prevalence of both of these conditions in recent years has caused them to become epidemic [1,2]. Indeed, T2D and OP share many risk factors, including non-modifiable factors such as aging and genetic predisposition; and modifiable factors such as lifestyle, diet, and physical activity; therefore, these two conditions very often coexist [1].

Due to increased life expectancy the number of diabetic patients worldwide has been steadily increasing over the years; in particular, T2D accounts for more than 90% of all diabetes cases worldwide [3]. Especially for long durations of disease, diabetes results in macrovascular (stroke, heart attack, and other cardiovascular diseases) and microvascular (nephropathy, neuropathy, and retinopathy) damage, all of which are responsible for increased morbidity and mortality [4]. OP is a common disorder of bone metabolism, characterized by low bone mass and microarchitectural deterioration of bone tissue, leading to bone fragility, with an increasing susceptibility to fractures [5,6]. This disorder can be related to several conditions and affects both sexes, with a higher incidence in women after menopause [5].

As reported in numerous studies, T2D and OP are closely related [1,2,7], and this association is not casual, since T2D subjects, similarly to patients with type 1 diabetes (T1D), have a higher risk of fragility fractures than the general non-diabetic population, as now demonstrated by several epidemiological observations [8,9,10]. The mechanisms underlying skeletal fragility in T2D are complex and not completely understood [7,11,12]. However, over the years, several studies have highlighted the contribution of hyperglycemia, inflammation, oxidative stress, bone marrow adiposity, changes in collagen properties, and alterations in bone cells function in T2D-induced bone fragility. As occurs for other diabetic complications, a key role in the development of impaired bone strength is likely played by hyperglycemia and the accumulation of advanced glycation end-products (AGEs) [2,7,11].

Although T2D and OP as single entities are per se responsible for increased all-cause mortality, different studies have reported a further increase in mortality in T2D subjects following a fragility fracture, compared with non-diabetic subjects with fractures, as well as with diabetic patients without fractures. T2D individuals who have suffered a hip or vertebral fracture have been shown to have an almost threefold increased risk of death [13,14].

Bone mineral density (BMD) and the WHO Fracture Risk Assessment (FRAX) algorithm are common tools that are generally used to assess fracture risk in the general population [5]. Although their importance in the clinical management of OP is well-known, their use in T2D may result in an underestimation of the fracture risk [15,16]. Importantly, T2D patients often present elevated or normal BMD values compared with non-diabetic or T1D subjects [14], including those cases reporting a fragility fracture. The limited reliability of these tools suggests the existence of specific mechanisms underlying bone fragility in T2D that are different from those in other forms of OP. Thus, hyperglycemia-induced changes in bone strength, long-term disease complications, or other comorbidities, as well as some antidiabetic drugs, may all adversely affect bone health and contribute to impaired bone quality and increased fracture risk in T2D [2].

**Figure 1:**
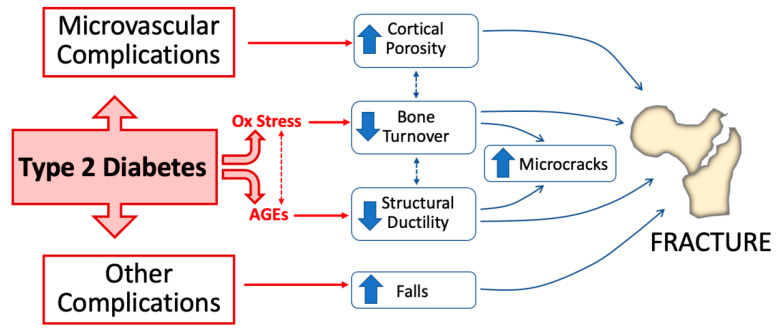
Pathophysiological mechanisms of bone fragility in type 2 diabetes. Alteration of glucose homeostasis in type 2 diabetes (T2D) leads to the accumulation of increased advanced glycation end-products (AGEs) in bone and enhances oxidative stress. Both of these conditions directly impact bone health by decreasing bone turnover and impairing structural bone ductility. Microvascular disease and other complications (e.g., neuropathy, retinopathy, and nephropathy) concur in T2D-induced bone fragility by, respectively, increasing cortical bone porosity and increasing the risk of falls.

This review mainly focuses on the role of AGEs and oxidative stress as major mechanisms underlying skeletal fragility in T2D (Figure 1) and provides some indications on how to improve fracture risk prediction in T2D patients.

## 2. Advanced Glycation End-Products and Bone Fragility

AGEs, together with their receptor (RAGE), a member of the immunoglobulin super-family transmembrane proteins, play a relevant role in the pathogenesis of diabetic osteopathy. AGEs are nothing but the products of nonenzymatic glycation of macromolecules. Many processes can lead to their formation, including a high-fat diet, oxidative stress, and a prolonged state of hyperglycemia. The formation of AGEs in the diabetic subject mainly relies on the Maillard reaction that begins with the combination of a carbonyl group of a reducing sugar or aldehyde with lysine, arginine, or amino-terminal residues of proteins, as well as with amino groups in lipids. Two other mechanisms besides this non-enzymatic reaction are the polyol pathway and lipid peroxidation [17,18]. Both experimental studies in vitro or in vivo (using different mice models of diabetes) and human studies indicate that AGEs have the capacity to accumulate inside the bone, altering bone turnover, the bone matrix composition, and ultimately bone strength [19,20]. In particular, the long half-life of type 1 collagen, the main organic constituent of bone, makes this protein highly susceptible to glycation and the formation of AGEs [21]. Less information is available about the possible implication of glycation of other proteins of the bone matrix on bone strength. In normal bone, enzymatic collagen crosslinks, such as deoxypyridinoline and pyridinoline, increase collagen fibril stiffness, thus enhancing skeletal strength. Conversely, with aging, and particularly in diabetes, glycation and/or oxidation generate irreversible non-enzymatic collagen crosslinking, ultimately impairing bone strength. However, skeletal AGEs not only include crosslinking modifications of collagen fibers but also non-crosslinking modifications (e.g., carboxy-methyl-lysine and carboxy-ethyl-lysine) [22].

### 2.1. In Vitro Cell-Based/Mouse Studies

In vitro studies demonstrated that glycation of bone specimens increases AGE content, and this likely occurs at different rates in cortical rather than trabecular bone [23,24]. Moreover, there is generally a negative relationship between AGE levels and post-yield bone mechanical properties [19,20]. Consistent with these data, glycation of human bone in vitro led to increased microcrack formation with mechanical stress, and increased levels of AGEs were identified in skeletal regions with microcrack damages [19].

In some but not all diabetic mice models, increasing AGEs and abnormal collagen glycation have been directly associated with a reduction in bone strength, without significant variation in BMD [20,21]. Differences among studies might be dependent on the used mice models; on the onset, degree, and duration of hyperglycemia relative to control animals; on the selected age for euthanasia; and on the different techniques to assess skeletal AGEs. In a very recent study using a diet-induced T2D model, a higher accumulation of AGEs (measured by biochemical assay and confocal Raman spectroscopy) was demonstrated in diabetic animals that was strictly related to the loss of fracture toughness [25]. Of interest, the use of phenacyl thiazolium chloride for the in vitro removal of glycation products partially rescued bone toughness.

In different tissues, including bone, AGEs may also exert indirect deleterious effects through their interaction with RAGE on the cell membrane. Indeed, AGE-RAGE binding underlies the pathogenesis of diabetes-induced damage at the endothelial level, particularly with the classic micro- and macro-vascular complications. AGEs themselves are able to stimulate the synthesis and exposure of RAGEs on the cell membrane through the activation of specific intracellular signaling pathways. Importantly, AGEs accumulate in the bone with age and/or T2D and come into close contact with osteoblasts or osteoclasts, which also express RAGE. AGE-RAGE-mediated tissue damage occurs via the activation of inflammation and oxidative stress itself, as well as by the activation of intracellular pathways, including PI3K/Akt, JAK/STAT, and others that cause reactive oxygen species (ROS) production. Regarding bone cells, AGE-RAGE binding mainly results in the altered differentiation and apoptosis of osteoblasts, with the upregulation of RANKL, leading to increased osteoclastogenesis and impaired bone mineralization [26,27,28,29]. In addition to the increase in RANKL mRNA expression, these effects are at least in part mediated by the downregulation of alkaline phosphatase and osteocalcin mRNA, as well as by an upregulation of RAGE expression that increases the AGE-RAGE pathway. AGE-RAGE binding also results in increased expression and secretion of TGF-beta, inhibiting the differentiation and mineralization of osteoblastic cells [28]; it also causes the suppression of signaling pathways that are important for skeletal homeostasis such as Wnt/β-cathenin (the master regulator of osteoblast formation and activity), PI3K, and ERK. Thus, albeit the exact mechanisms are not completely understood, AGE-RAGE interaction in bone cells leads to decreased osteoblast function, impairs bone mineralization, and likely increases the osteoclast number; however, the ultimate effects on osteoclast function remain to be established [19]. A single in vitro study on osteocytic cell lines indicated that AGEs significantly decrease RANKL and increase sclerostin expression (a major negative regulator of the Wnt/β-cathenin pathway) [30], and this is somewhat consistent with the report of low bone turnover frequently described in T2D patients.

**Figure 2:**
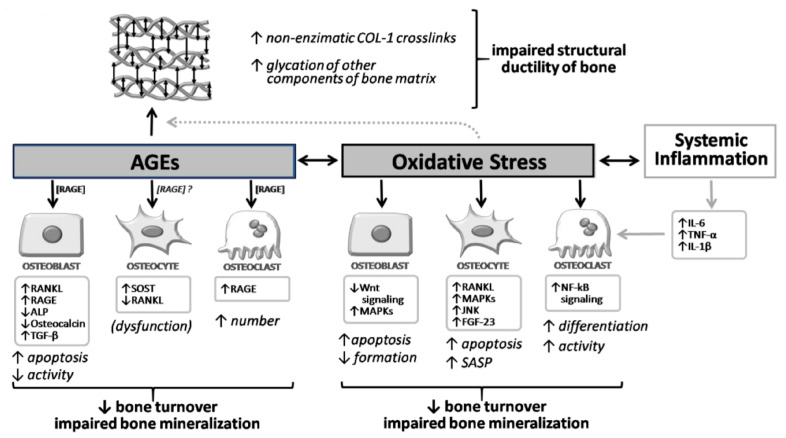
Overview of the molecular and cellular effects of AGEs and oxidative stress on bone. AGEs and oxidative stress exert direct effects on osteoblast, osteocyte, and osteoclast. Moreover, AGEs also increase non-enzymatic crosslinks of collagen type 1 and the glycation of other components of the bone matrix, impairing the structural ductility of bone. This process isenhanced by oxidative stress conditions. ↓, decrease; ↑, increase.

Moreover, we must also consider that other ligands can bind to RAGE (such as HMGB1 produced by myeloid cells, osteoblasts, osteoclasts, and bone apoptotic cells), resultingin increased levels of RANKL, TNF-alpha, and IL-6 in osteoblasts themselves and in stromal cells [31]. Thus, the binding of RAGE with other ligands appears to be also involved in the regulation of osteoclast activity. It also seems that RANKL stimulates RAGE expression and, consequently, osteoclast differentiation [31,32]. Studies on the silenced RAGE gene have demonstrated an attenuation of RANKL-mediated osteoclastic differentiation [33], with reduced bone resorption, reduced osteoclast number, and increased bone mass [34]. Importantly, RAGE-AGE interaction also results in a detrimental effect at the level of progenitor cells, and particularly the bone marrow stromal cells (BMSCs) [35], so that the inhibition of RAGE signaling is important for maintaining BMSCs in vitro and could favor their differentiation into adipocytes, osteoblasts, and osteocytes. The role of RAGE, therefore, is central for most of the complications of diabetes, including diabetic osteopathy. Nevertheless, it appears that the part of the signals induced by RAGE-AGE binding may also have a positive role on skeletal health by mediating the anabolic effect of PTH on bone [36]. A summary of the molecular and cellular effects of AGEs in bone, along with their interaction with oxidative stress, is given in Figure 2.

### 2.2. Clinical Studies

As suggested by preclinical data, AGEs may accumulate within the tissues of diabetic patients, including bone, altering matrix properties, bone turnover, and thus bone strength, regardless of BMD (Figure 1 and Figure 2). As mainly demonstrated in the animal models, AGEs directly impair enzymatic crosslinking while increasing non-enzymatic crosslinks in bone collagen. Such an abnormal collagen glycation negatively impacts the material and biomechanical properties of cortical and cancellous skeletal compartments, thus allowing microdamage to spread more easily and making bone tissue more fragile and more likely to fracture [21].

To date, a detailed characterization of the AGE content in bone from patients with T2D has not been performed, albeit the available information suggests that pentosidine levels (a non-enzymatic collagen crosslink) in bone specimens of T2D patients are 20–30% higher than in non-diabetic subjects [19,37]. Moreover, it seems that the age-related increase in AGEs that occurs earlier and at a higher amount in T2D is more easily detected in cortical than trabecular bone [24,38]. Such a limited information is, above all, related to the difficulty of obtaining a precise and comprehensive dosage of the AGE content in human bone. In fact, a direct, invasive assessment of bone specimens is required. Moreover, most of the research performed to date primarily measured pentosidine or total fluorescent AGEs (through bulk fluorescence of hydrolysates of bone) that only represent a limited proportion of skeletal AGEs. In fact, non-fluorescent crosslinks (e.g., glucosepane) or AGEs involving the modification of the side chain of a protein or lipids have not been considered [19,20]. Thus, few studies have directly assessed pentosidine or, less frequently, total fluorescent AGEs in human bone specimens obtained from biopsy, osseous material derived from orthopedic surgery, or cadaveric bones. These studies were generally consistent with animal studies and suggested a negative relationship between skeletal AGE content and both bone material properties and bone turnover, either in diabetic or non-diabetic subjects [20].

As a surrogate of skeletal AGEs, levels of urinary ad serum pentosidine or carboxy-methyl-lysine (a nonfluorescent AGE that accumulates at much higher levels in bone than pentosidine with aging and in diabetes), as well as skin autofluorescence, have been considered in clinical observational studies of large patient cohorts. Of interest, increasing levels of pentosidine (measured in serum or urine) or carboxy-methyl-lysine have been both associated with a higher risk of fractures in T2D patients [39,40,41,42]. In the largest of these studies, performed in the Health, Aging, and Body Composition prospective cohort of older adults, serum carboxy-methyl-lysine levels were remarkably higher in T2D than non-diabetic patients and were significantly associated with a higher risk of incident clinical fracture only in T2D cases (HR 1.49; 95%CIs, 1.24–1.79, per 1-SD increase in log carboxy-methyl-lysine) [42]. Likewise, in a group of postmenopausal women with T2D, the accumulation of AGEs, indirectly assessed with skin autofluorescence, was associated with a decrease in bone material strength index, measured by reference point indentation [43]. In fact, type 1 collagen, the major target for AGE accumulation in the skeleton, is also abundant in the skin, and some studies have demonstrated that skin and bone pentosidine levels per milligram of collagen are strongly correlated [44], making skin autofluorescence a likely surrogate marker of fluorescent skeletal AGEs for clinical use. Indeed, nonfluorescent AGEs such as carboxy-methyl-lysine interact differently with bone collagen with respect to fluorescent AGEs such as pentosidine, since they do not form intermolecular crosslinks within the organic matrix of bone, but due to a negatively charged carboxyl group, they can attract positively charged calcium ions and modify the charge distribution of proteins such as collagen, thus altering the molecular organization of the extracellular matrix [45,46]. This can alter bone mineralization and energy dissipation, both of which are known to negatively affect bone strength [47].

In summary, while it is plausible that increased AGE content in bone impairs bone strength, a gold-standard measure of skeletal AGEs for clinical use has yet to be defined, and additional prospective studies in large samples of T2D are required to definitely assess the impact of AGEs on the bone fragility of diabetic and non-diabetic subjects. After that, clinical trials should be finally required to determine whether the use of compounds inhibiting skeletal accumulation of AGEs is effective in preventing fragility fractures.

## 3. Oxidative Stress, Diabetes, and Bone Fragility

Diabetes mellitus induces oxidative stress through several mechanisms, including the polyol pathway, the glycation reaction with increased production of AGEs, a protein kinaseC-dependent activation of membranous NADPH-oxidase, and the mitochondrial electron transport chain [48,49,50,51]. In fact, hyperglycemia/hyperinsulinemia and impaired fatty acid metabolism in diabetes promote the exacerbation of oxidative processes and, ultimately, the production of free radicals and other ROS [50,51,52]. This particularly occurs in the mitochondria of several cell systems, leading to a wide range of systemic complications, which also include bone fragility and a high risk of fractures [53].

Different experimental studies in vitro and in vivo suggested that oxidative stress affects osteoblast, osteocyte, and osteoclast, causing an unbalance between bone formation and bone resorption (in favor of bone resorption) and impairing bone mineralization [53,54] (Figure 2). In more detail, an increase in ROS has been directly related to an impaired osteoblast/osteoclast balance, characterized by the inhibition of osteoblast differentiation and maturation, increased apoptosis of osteoblast and osteocytes, and an increase in osteoclast activation and activity, primarily driven by the RANK/RANK-ligand/NF-κB pathway [55,56]. Furthermore, several pieces of evidence underlined the connection between oxidative stress and bone impairment via the activation of peroxisome proliferator-activated receptor gamma (PPARγ), a nuclear hormone receptor stimulated by different ligands, including oxidized lipids and cytokines [57,58,59,60,61]. The activation of the PPARγ pathway, in particular, impairs Wnt/β-catenin signaling. This enhances osteoblast and osteocytes apoptosis and promotes adipogenesis at the expense of osteogenesis, thereby altering bone marrow homeostasis and, ultimately, bone quality [61]. In fact, oxidative stress and ROS decrease the proliferation of bone marrow mesenchymal stromal cells, thus reducing osteoblast precursors [54]. At the same time, the use of natural antioxidants seems to prevent and/or reverse these negative effects on bone cells. In agreement with these data, in mouse models, the loss of bone mass appears to be inversely related to ROS and glutathione reductase (GSR) activity in the bone marrow, as well as to reduced osteoblast formation and maturation due to glutathione inhibition [62,63,64].

Importantly, mice models of diabetes develop low bone turnover osteopenia and bone fragility with increasing glucose levels that, in some studies, have been associated with increased oxidative stress [65,66,67]. In a first model, in streptozotocin-induced diabetic mice (a T1D model), which exhibit a low turnover condition of bone fragility, the urinary excretion of 8-hydroxydeoxyguanosine, a marker of oxidative DNA damage, was elevated, and intensified immunostaining of an oxidative stress marker was observed in bone, and particularly in the osteoblasts, of diabetic mice [65]. Similar results were observed in a different model, the non-obese type 2 spontaneously diabetic Torii, where, of interest, an increased level of 8-hydroxydeoxyguanosine was observed, together with a decrease in the mineral apposition rate and the bone formation rate per bone surface [66]. In that model, all of these abnormalities (including the increase in 8-hydroxydeoxyguanosine) were completely prevented by insulin therapy and the normalization of glucose levels. These findings have also been confirmed in human studies, where a correlation between oxidative stress and reduced BMD was found, together with a beneficial effect of antioxidants on bone turnover and bone loss [68,69,70], irrespective of diabetes status. Indeed, the relative increase in osteoclast over osteoblast activity typically observed in postmenopausal osteoporosis may be at least in part associated with an imbalance between oxidant and antioxidant status, as a consequence of the decrease in estrogen levels [53]. In keeping with these observations, a prospective analysis in the Nurses’ Health Study cohort of 996 postmenopausal women, plasma fluorescent oxidation products (generated from many different pathways and reflecting the global oxidation burden) were positively associated with the risk of incident hip fractures [71]. However, information about oxidative stress markers and bone fragility in patients with T2D is very limited. In a small study on postmenopausal T2D women, a 50% reduction of circulating osteogenic precursors (a sort of circulating preosteoblast that may access bone-formation sites through blood vessels) was observed, and these cells had an increased expression of oxidative stress marker p66(Shc) and ofthe antioxidant defense enzyme superoxide dismutase, a target of the *Fox01* transcription factor that is activated in response to oxidative stress [72]. These findings were consistent with the reduced indices of bone formation, including mineralizing surface, osteoblast surface, and bone formation rate, as assessed by histomorphometry in a subgroup ofbiopsied T2D women recruited in that study.

Phospholipids containing polyunsaturated fatty acids, which constitute integral components of all cellular membranes, are also affected by oxidative stress, since they are susceptible to the lipidperoxidation caused by ROS [73]. Lipid peroxidation generates highly reactive degradation products such as malondialdehyde, 4-hydroxynonenal, and oxidized phospholipids such as oxidized phosphatidylcholine that are able to react with amino groups on proteins and other lipids to form the so-called oxidation-specific epitopes. Such oxidized phospholipids are common in many inflammatory conditions and are present on the surface of apoptotic cells and oxidized low-density lipoproteins (OxLDLs). An increased lipid peroxidation also occurs in diabetes, and a large body of clinical and experimental evidence indicates this process as a main cause of the metabolic and hemodynamic abnormalities associated with the increased cardiovascular risk in diabetic subjects [74].

Indeed, compared with their respective age-matched controls, diabetic patients had greater oxidative damage to lipids and proteins, as demonstrated through the analysis of circulating hydroperoxides, lipoperoxides, and oxidation protein products [75,76]. Of interest, a relationship between lipid peroxidation and bone fragility has been more recently demonstrated. Evidence suggests that these bioactive molecules induce bone loss in mice by inhibiting the differentiation of osteoblasts and promoting the differentiation of osteoclasts [77]. In the ApoE-null mice model fed a high-fat diet (HFD), together with atherosclerosis, the activation of vascular inflammation by OxLDL led to a dramatic reduction in osteoblast number and function at either trabecular or cortical bone sites, whereas the osteoclast number was modestly reduced only in trabecular bone [78].

A decrease in osteoblast progenitors was also demonstrated. These effects were related to a downregulation of Wnt signaling, leading to a reduced expression of Wnt pro-osteoblastogenic target genes, together with an increase in the number of monocyte/macrophages in the bone marrow and an increased expression of inflammatory cytokines such as IL-1β, IL-6, and TNF. Likewise, OxLDL attenuated osteoblast formation and induced osteoblast apoptosis in vitro [79,80,81]. These oxidation-specific epitopes are members of a larger group of proinflammatory and immunogenic molecules that are produced by excessive oxidative stress (as it occurs during inflammatory conditions or disorders such as T2D). Their negative effects on the vasculature and bone can be prevented through specific evolutionary conserved pattern-recognition receptors that can be either cell bound (such as the large family of scavenger receptors and toll-like receptors) or soluble, such as the natural antibodies produced by B-1 lymphocytes [73,82,83,84]. In this regard, the neutralization of oxidized phospholipids through the natural B-1 lymphocyte IgM antibody E06 attenuated high-fat-diet-induced bone loss in a mice model by increasing the osteoblast number and stimulating bone formation, likely through a Wnt-pathway-mediated mechanism [85,86,87]. Importantly, experimental evidence also suggested that these bioactive lipids can blunt the effects of bone anabolic agents, such as teriparatide (PTH 1–34), acting through the protein kinase A, as well asWnt and/or IGF-I-dependent mechanisms [88,89,90,91]. Consistent with these preclinical observations, in patients receiving teriparatide, lumbar BMD changes were negatively correlated with total cholesterol and positively correlated with HDL cholesterol [92].

In line with all the findings described above, several experimental observations demonstrated that the use of antioxidants can prevent the inhibition of osteogenic differentiation, the osteoblast apoptosis, and the impaired mineralization process due to oxidative stress [93,94,95]. One of these studies specifically investigated the in vitro effects of Gomisin A, a natural compound with antioxidant properties isolated from fruit extract, on osteoblast differentiation under high-glucose-induced oxidative stress. This compound potentially regulated osteoblast differentiation despite the high oxidative stress condition, via the upregulation of heme oxygenase-1 and maintenance of mitochondrial homeostasis [94]. Consistent with these findings, the overexpression of human Thioredoxin-1 (a major intracellular antioxidant) in transgenic mice prevented the increase in oxidative stress markers such as 8-hydroxydeoxyguanosine, partially restored the reduction in BMD, and prevented the suppression of bone formation [96].

At the same time, a persistent enhanced oxidative state appears related to an increased risk to develop both T1D and T2D, due to the lack of pancreatic β-cells’ intrinsic defensive mechanisms. Accordingly, a large class of proteins, FoxOs, plays an important role in maintaining cytoplasmatic balance and reducing intracellular oxidative stress via several pathways, in particular, PI3/Akt activation, stimulating β-cells proliferation and survival, but also inducing apoptosis when oxidative damage is irreparable [88,97]. Indeed, oxidative stress and FoxO are closely related, even at the bone level. ROS induces increased transcription of FoxO, and this, in turn, contributes to the reduction of the effects of oxidative stress on bone cells. In addition, oxidative stress induces the association between FoxOs and beta-catenin, thus causing a reduction in osteoblast differentiation via the inhibition of the Wnt/beta-catenin and T-cell factor pathway [98,99,100]. Moreover, as previously underlined, PPARγ acts as an inhibitor of osteoblastogenesis and promotes adipogenesis. Of interest, the production output of PPARγ is directly suppressed by an increase in FoxOs expression [101,102]. In agreement with these data, in mouse models with the deletion of the genes encoding for FoxO subclasses, a substantial increase in ROS was found in the bone matrix, causing an increase in apoptosis of osteoblasts and osteocytes and, ultimately, a reduction in total bone mass, particularly in mice with triple deletion of FoxO genes (FoxO1, -2, and -3) [103]. ROS can induce an inhibition of FoxO through direct phosphorylation via the Akt pathway. In addition, ROS can directly stimulate NF-κB by causing the inhibition of FoxO, particularly FoxO3 [104,105].

Finally, diverse forms of age-related stress or metabolic insults, which also include ROS and oxidative stress, may all converge to cause cell senescence in different tissues, including bone [106,107]. Thus, the increase in oxidative stress in T2D may contribute to the development of an altered gene-expression profile within different cell systems that includes the upregulation of anti-apoptotic pathways and a senescence-associated secretory phenotype (SASP) consisting of pro-inflammatory cytokines, chemokines, and matrix remodeling proteins, both of which represent the hallmark of cellular senescence. This also occurs in bone with ageing and diabetes [107]. Indeed, in a recent study involving a non-genetic mouse model mimicking human adult-onset T2D, together with increased AGEs and activation of the RAGE signaling pathway, a premature accumulation of senescent osteocytes with a unique proinflammatory signature was demonstrated [108].

## 4. Other Mechanisms of Bone Fragility in Type 2 Diabetes

While the increases in oxidative stress and AGE deposition represent a major direct mechanism to explain the skeletal alterations leading to T2D-induced bone fragility, other additional factors have been implicated (Figure 1). For example, diabetic complications such as neuropathy, microvascular damage, and/or retinopathy represent a main cause of falling and, thus, fall-related fractures, especially in elderly T2D patients [109,110]. Likewise, nephropathy represents an additional and severe complication of T2D that further negatively impacts bone turnover (often leading to hyperparathyroidism and the so called “adynamic bone disease”) and, ultimately, bone strength [111]. Moreover, obesity is often associated with T2D, with different and somewhat contrasting implications for bone turnover, BMD, and bone fragility, as well as for inflammation and oxidative stress [112].

Indeed, most, if not all, of these complications are also strictly connected to oxidative stress and increased AGE accumulation in the different target tissues. Moreover, the microvascular complications of T2D might also be directly implicated in the impairment of bone quality of T2D. It has been established that the crosstalk between bone and vessels is essential for optimal bone development, as well as for proper function and repair after fracture [113]. In this respect, recent findings indicate that, after a fracture, in T2D patients, there is an increased risk of nonunion or delayed union, as well as impaired fracture healing [114]. Importantly, mesenchymal stem cells have an intrinsic osteogenic capacity and promote vascularization by communicating with endothelial cells through proangiogenic factors such as VEGF, IGF, PDGF, and FGF [115]. A study performed in 2020 showed that, within a group of patients with T2D, those with microvascular damage, as documented through reduced transcutaneous oxygen tension (<40 mmHg), had increased cortical porosity at the level of the distal tibia, and this per se represents a risk factor for fractures [116]. Consistent with these data, an increase in cortical porosity, as assessed by high-resolution peripheral QCT, was found in T2D patients with microvascular disease [117], and particularly in T2D cases with fragility fractures, as compared with non-fractured T2D patients [118,119,120]. It has been thus proposed that, in T2D, the accumulation of AGEs is mainly responsible for the impairment of bone material properties, while microvascular disease causes an increase in cortical porosity, with both of these conditions representing independent mechanisms of bone fragility [7].

In addition, insulin treatment and the relatedhypoglycemic events are additional cofactors in increasing the risk of fall and fractures in T2D [121,122]. Indeed, with insulin treatment generally being reserved for advanced disease status, after failure with oral antidiabetic agents, these patients also have an increased risk of developing most T2D-related complications, including bone fragility.

Finally, sarcopenia could play an important role in T2D bone fragility, thus further impairing bone quality and eliciting a higher risk of fall in diabetic individuals [123]. However, limited data are available to date about this association, and the results may be somewhat altered because of several confounding factors, in particular, the neuromuscular dysfunction often inherent in diabetics individuals [124,125,126]. Moreover, several studies showed low serum vitamin D levels and their inverse correlation with HbA1c levels in diabetic subjects, even after adjusting the results for confounding factors (such as body mass index) [127,128]. Deficiency in serum vitamin D might thus contribute not only to impaired glucose tolerance but also to sarcopenia and impaired bone quality in T2D subjects, therefore increasing the risk of falling and fractures [129,130].

## 5. Assessment of Fracture Risk in Patients with Type 2 Diabetes

**Figure 3:**
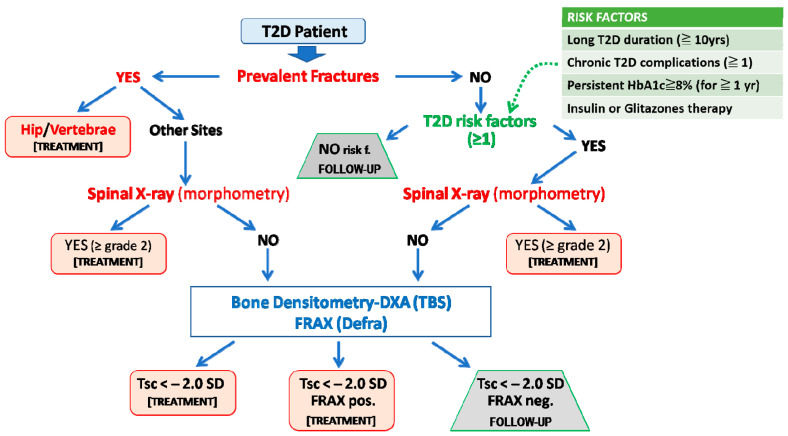
Algorithm for fracture risk stratification in type 2 diabetes. The prediction of fracture risk in patients with type 2 diabetes (T2D) should first rely on the presence of fragility fractures (including the identification of morphometric vertebral fractures) or, in their absence, on T2D-specific risk factors (e.g., duration of disease, glycemic control, ongoing treatments with insulin or thiazolidinediones, and the presence of typical T2D complications). Then either BMD or FRAX assessment could be used as an additional informative tool. We suggest that FRAX should be country-adapted and calculated without bone mineral density and with rheumatoid arthritis as a surrogate risk factor of diabetes (FRAXpos = patients who fulfill the National Osteoporosis Foundation criteria for treatment: 20% ten-year risk of major fragility fractures and 3% ten-year risk of hip fracture). Adapted from Reference [2].

As previously outlined, and due to the peculiar mechanisms that have been implicated in bone fragility in T2D, the identification of patients at high risk of fracture is a challenging task. In fact, the measurement of BMD or the use of algorithms such as FRAX often underestimates fracture risk in patients with T2D [15,16]. Thus, the presence of a previous fragility fracture, together with specific risk factors associated with increased fracture risk in T2D, represents the initial information to be considered for the stratification of fracture risk in these patients, as recently outlined by some position statements [2,131,132]. A representative flowchart summarizing the outcomes from one of these documents [2] that is also, in part, consistent with a previous report from a working group from the International Osteoporosis Foundation [131] is shown in Figure 3.

In the algorithm, the first factor to consider is the presence of past fractures. If there is a prior fracture at a typically osteoporotic site (e.g., hip or vertebrae), the T2D patients should be considered for anti-osteoporotic treatment regardless of the other factors. In the case of fractures at other sites, the presence of vertebral fractures should be then evaluated through spinal X-ray or morphometric evaluation during the DXA assessment. In the case of one or more moderate/severe (grade 2–3, according to the Genant semiquantitative visual approach [133]) vertebral fractures, an active treatment should be considered. If no fractures are present, we need to consider the presence of common risk factors or comorbidities associated with osteoporosis (e.g., use of glucocorticoids, tobacco, and alcohol abuse), as well as specific risk factors directly related to T2D. These are generally related to the duration, control, and treatment of diabetes and mostly reflect the persistence of high-risk conditions for bone fragility in these patients due to uncontrolled hyperglycemia and its negative effects on the skeleton and/or in other target organs.

Four main risk factors were considered in the flowchart reported in Figure 3. The first of these factors is the duration of diabetes. In fact, many studies highlighted how a long duration of disease can result in detrimental skeletal effects through the already described mechanisms of glucose toxicity (AGEs), oxidative stress, and microvascular damage. In a large report from the Blue Mountains Eye study (including 3654 T2D subjects), a disease duration longer than 10 years was associated with an increased risk of all osteoporotic fractures (3.3; 95%CIs, 1.3–8.2) and of proximal humerus fracture (11.4; 95%CIs, 2.4–54.2) [134]. A subsequent retrospective study in a Canadian cohort (82,094 diabetic adults and 236,682 controls) showed that in subjects with T2D for more than 5 years, there was an increased risk of both fracture at all osteoporotic sites (1.15–95% CIs, 1.09–1.22) and hip fractures (1.4–95% CIs, 1.28–1.53) as compared to T2D patients with a shorter duration of disease [135]. Based on these data and on other similar observations, a T2D duration of 5 [131,132] or 10 years [2] was suggested in the position statements. In this respect, however, it should be remarked that T2D often remains a misdiagnosed disease for several years. Another important risk factor is the presence of systemic complications related to diabetic disease, such as retinopathy, nephropathy, neuropathy, and cardiovascular damage. Indeed, microvascular damage, as already pointed out, also occurs at the bone level, with increased cortical porosity, thus resulting per se in increased risk of fracture [7]. In this respect, a case-control study of 124.655 fractured patients and 373.962 non-fractured controls showed that diabetes and all of its complications are associated with an increased risk of fractures [136]. Although the specific weight of each factor on fracture remains unknown, it has been highlighted that, in addition to cardiovascular or renal complications, diabetic retinopathy or neuropathy is also directly and independently associated withthe increased risk of fractures [134,137]. Furthermore, poor glycemic control can result in an elevated risk of fracture, regardless of the duration of the disease, as also evidenced by the Rotterdam Study, in which, within a sample of 4.135 diabetic subjects with an average follow-up of 12.2 years, a 1.6-fold increased risk of fracture was shown in individuals with poorly controlled diabetes compared to those with good glycemic control [138]. Given the relative contribution of a single measurement of fasting glucose or HbA1c levels as a risk factor for bone fragility in T2D (since persistent rather than transient hyperglycemia is considered to be a relevant condition that negatively affects bone health), it has been suggested that only those cases with persistently impaired glucose control (e.g., HbA1c levels above 7.5–8% for at least 1 year), irrespective of T2D duration, or the presence of complications, should be also be considered at risk of fracture [2,131,132]. Finally, some antidiabetic drugs such as thiazolidinediones or insulin have been related to an increased risk of fractures in diabetes and should be thus considered as additional risk factors [2,131,132]. The negative effects of thiazolidinediones on skeletal health are well-known and are strictly related to their inhibitory effect on PPAR-γ [139,140]. For what concerns insulin therapy, although its anabolic effect is well-known at the bone level in experimental conditions, its association with increased fracture risk can be due to the high risk of hypoglycemic events predisposing to falls, as well as to the preeminent use of this therapy for long-standing diabetic subjects with poor glycemic control. Overall, the presence of at least one of the abovementioned specific risk factors for fracture in T2D patients without prevalent fractures indicates the need for further diagnostic investigations. The latter first consists in excluding the presence of previously undiagnosed morphometric grade 2–3 vertebral fractures that per se give the indication of active anti-osteoporotic treatment. In the case that prevalent and morphometric vertebral fractures have been excluded in a patient with at least one of the T2D specific risk factors for fracture, either a DXA analysis or the assessment of FRAX score should be advised.

However, as previously shown, the use of the classic FRAX algorithm can result in an underestimation of fracture risk in these patients, so that some adjustments have been recommended [141]. These include the selection of rheumatoid arthritis as a proxy comorbidity for T2D (that is not included in the algorithm), the reduction of the femoral neck T-score by 0.5 SD, or the increase of the subject’s age by 10 years. All of these adjustments, however, are able to slightly improve fracture risk prediction by still underestimating the effective risk. Based on a recent preliminary observation, when using the algorithm of Figure 3, a practical approach could consist in using the rheumatoid arthritis adjustment, without including the BMD information [2,142]; however, this remains to be universally validated in large patient cohorts.

Concerning BMD assessments by DXA, all the position statements suggest the use of a different threshold, that is, −2.0 SD or below, instead of the −2.5 SD generally considered for the diagnosis of osteoporosis in the general population, since T2D patients classically fracture at higher BMD values [2,131,132]. Thus, combining BMD and FRAX information in T2D patients without fractures, but with at least one specific risk factor for fractures, can be of help for treatment decision-making in the case of a T-score < −2.0, regardless of FRAX results, or with a T-score > −2.0 in the presence of FRAX, suggestive of a high fracture risk. The latter corresponds to estimates provided by the National Osteoporosis Foundation criteria, namely 20% ten-year risk of major fragility fractures and 3% ten-year risk of hip fracture [143]. Instead, the presence of T-score values > −2.0, together with a low risk FRAX score, excludes the necessity of treatment and suggests a periodic follow-up.

Potential tools to be investigated and eventually integrated into the diagnostic approach for a better stratification of fracture risk in T2D include the assessment of AGE status (e.g., pentosidine, carboxy-methyl-lysine, or skin autofluorescence), diagnostic techniques (e.g.,high-resolution peripheral QCT or magnetic resonance), or other ways to estimate bone quality and strength (e.g., trabecular bone score and bone microindentation) [2,119,144,145,146,147,148] (Table 1).

**Figure 4:**
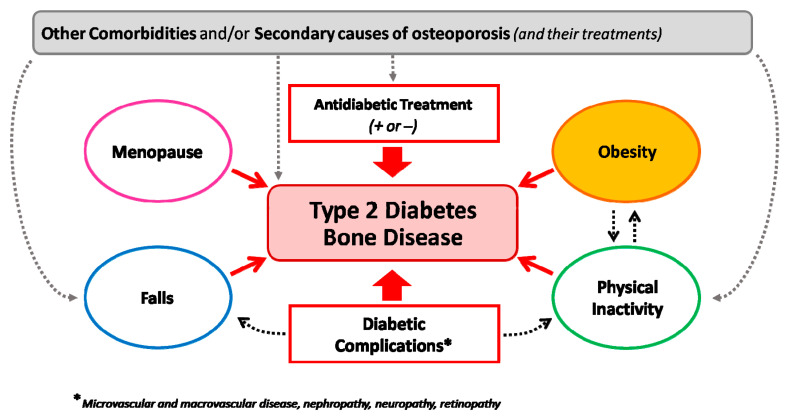
Heterogeneous determinants of bone fragility in type 2 diabetes. The figure underlines the interaction among different conditions interacting with type-2-diabetes-induced bone fragility on enhancing the risk of fractures in different patient settings.

Of course, when assessing fracture risk in a patient with T2D, we should also consider the presence of other conditions that may add to diabetes in regard to altering bone strength and quality, such as obesity, menopause, the use of glucocorticoids, and comorbidities (i.e., BCO or rheumatic diseases) (Figure 4). The presence of one or more of these conditions might in fact add to the direct negative effects of T2D on bone and eventually change the clinical picture (e.g., leading to an increased bone turnover and/or bone loss).

## 6. Conclusions and Future Directions

T2D and OP are common disorders of ageing with a relevant health burden. While T2D has long been considered neutral (if not protective) for bone health, mainly due to the reportof normal or high BMD levels in T2D patients, it has now been established from large-scale prospective observations that this disorder confers an increased bone fragility and a high fracture risk [8,9,10,11,12]. Moreover, morbidity and mortality following a fragility fracture are increased in T2D patients compared to the general population, even concerning vertebral fractures [13,14]. As it occurs for other complications of diabetes (e.g., microvascular disease, retinopathy, and neuropathy), the increases in AGEs and oxidative stress due to persistent hyperglycemia represent the major mechanisms directly affecting bone fragility in T2D. As shown in Figure 1 and Figure 2, both of these factors contribute to an impairment of structural ductility and other characteristics of bone quality, as well as to an alteration of osteoblast and osteoclast balance, often leading to decreased bone formation. Moreover, AGEs and oxidative stress have an additional negative impact on skeletal health, since they are also major determinants of microvascular disease, which has been related to an increase in the cortical porosity of bone [7]. Conversely, BMD is not much affected by these factors, making stratification of fracture risk in T2D more complex than in other forms of osteoporosis. In this respect, either fracture risk assessment tools (e.g., FRAX) or the measurement of bone turnover markers have a poor predictive value for the prediction of fracture risk in T2D. Thus, fracture risk stratification in T2D should first rely on the presence of fragility fractures (including the identification of morphometric vertebral fractures), as well as on specific risk factors, such as the duration of disease, glycemic control, ongoing treatments for T2D (e.g., insulin or thiazolidinediones), and the presence of typical T2D complications [2,131]. Then either a BMD or FRAX assessment could be used as additional informative tools. In this respect, there is an urgent necessity to improve fracture risk prediction with the use of additional diagnostic tools such as those involved in the assessment of AGE accumulation, DXA-derived approaches (i.e., TBS or HSA), HR-pQCT, and MRI that might add additional information (as summarized in Table 1). All of these approaches, however, need to be validated in large prospective studies.

## Figures and Tables

**Table 1:** Studies that analyzed additional diagnostic tools for the prediction of fracture risk in type 2 diabetes.

Tool	Parameter	Summary of Findings
**Measurement of AGEs** (Advanced Glycation End products)	Pentosidine	Increasing levels of pentosidine is associated with higher risk of fracture [39,40,41].
	Carboxy-Methyl-Lysine	Higher levels are associated with increased risk of incident clinical fractures in T2D, independent of BMD [42].
	Skin Autofluorescence	High skin autofluorescence was associated with a decrease in bone material strength index, measured by reference point indentation [43].
**HRpQCT** (High Resolution peripheral QCT)	Cortical Porosity	T2D subjects with fractures showed an increased cortical porosity (4.8-fold) as compared with T2D cases without fractures [118]. In particular, the increased cortical porosity is a characteristic of a subgroup of T2D subjects which presents microvascular complications [117].
**DXA derived**	TBS (Trabecular Bone Score)	TBS predicted major osteoporotic incident fractures in diabetic and non-diabetic subjects independently of BMD [2,144].
	HSA (Hip Structural Analysis)	Some studies suggest a weaker geometry and an impaired skeletal load response estimate in T2D [138,145,146]. These alterations seem to be more evident in T2D subjects with a worse glucose control and more severe disease [138].
**Microindentation**	BMSi (Bone Material Strength index)	Many studies demonstrate a reduction of BMSi in T2D post-menopausal women, before and after adjusting for covariates [43,119,147]. BMSi was also lowest in long-duration disease and in higher HbA1c levels.
**MRI** (Magnetic Resonance)	Trabecular network	T2D postmenopausal women have deficits in trabecular network at the distal radius, compared to controls [148].
